# Bacterial Proprioception: Can a Bacterium Sense Its Movement?

**DOI:** 10.3389/fmicb.2022.928408

**Published:** 2022-07-07

**Authors:** Rachit Gupta, Junhua Yuan, Pushkar P. Lele

**Affiliations:** ^1^Artie McFerrin Department of Chemical Engineering, Texas A&M University, College Station, TX, United States; ^2^Department of Physics, University of Science and Technology of China, Hefei, China

**Keywords:** mechanosensing, proton-motive force, CheY, chemotaxis, motility

## Abstract

The evolution of the bacterial flagellum gave rise to motility and repurposing of a signaling network, now termed the chemotaxis network, enabled biasing of cell movements. This made it possible for the bacterium to seek out favorable chemical environments. To enable chemotaxis, the chemotaxis network sensitively detects extracellular chemical stimuli and appropriately modulates flagellar functions. Additionally, the flagellar motor itself is capable of detecting mechanical stimuli and adapts its structure and function in response, likely triggering a transition from planktonic to surface-associated lifestyles. Recent work has shown a link between the flagellar motor’s response to mechanical stimuli and the chemotactic output. Here, we elaborate on this link and discuss how it likely helps the cell sense and adapt to changes in its swimming speeds in different environments. We discuss the mechanism whereby the motor precisely tunes its chemotaxis output under different mechanical loads, analogous to proprioception in higher order organisms. We speculate on the roles bacterial proprioception might play in a variety of phenomena including the transition to surface-associated lifestyles such as swarming and biofilms.

## Introduction

Propulsion by rotating flagella is among the dominant forms of motility in the bacterial kingdom. Rotation of the flagellum is enabled by a rotary device called the flagellar motor. Modulation of the direction or the speed of flagellar rotation can bias the cell’s migration in three-dimensional space (Dickinson and Tranquillo, 1993; Armitage, 1999; Attmannspacher et al., 2005; Wadhwa and Berg, 2022). Unsurprisingly therefore, many bacterial species modulate flagellar functions to swim toward favorable habitats (Silversmith and Bourret, 1999; Eisenbach, 2004). Migration up or down a gradient of extracellular ligands, known as chemotaxis, powerfully influences the likelihood of successful host invasion, colonization, and survival (Kollmann et al., 2005; Lertsethtakarn et al., 2011).

Chemotaxis is enabled by the coupling of a two-component signaling pathway and the flagella (Falke et al., 1997). Chemoreceptors detect changes in the concentration of extracellular ligands and respond by controlling the activity levels of a histidine kinase, CheA. In turn, CheA modulates the phosphorylation of CheY. CheY-P is a freely diffusible cytoplasmic molecule that interacts with the flagellar motor to modulate its function (Welch et al., 1993; Sarkar et al., 2010). In *Escherichia coli*, the dephosphorylation of CheY-P is accelerated by a phosphatase CheZ that localizes mostly at the receptors (Hess et al., 1988; Cantwell et al., 2003), while in some bacteria such as *Bacillus subtilis*, a structural component (FliY) within the motor itself contributes to CheY-P dephosphorylation (Szurmant et al., 2003). In many chemotactic species, multiple CheY homologues exist that exhibit complex interactions with the motor (Porter et al., 2006). In *E. coli*, a single CheY modulates flagellar switching: the binding of CheY-P to the base of the flagellar motor promotes clockwise (CW) rotation in an otherwise counterclockwise (CCW) rotating motor (Pan et al., 2017). Modulation of the directional switching gives rise to the run-tumble pattern of movement that forms the basis of chemotaxis in *E. coli*. Additionally, certain metabolites can interact directly with the motor to modulate directional switching independent of CheY (Yang et al., 2020; Gupta et al., 2022).

As the bacterium swims up or down a ligand gradient, the varying ligand concentration offsets the CheA activity from its basal value. This response could saturate the response unless CheA activity resets. The resetting, called adaptation, is mediated by two enzymes—a methyltransferase (CheR) and a methylesterase (CheB). CheR and CheB methylate and demethylate the receptors to *precisely* adapt the activity of CheA and thus of CheY-P levels (Parkinson and Kofoid, 1992; Armitage, 1999). Precise adaptation in CheY-P levels helps maintain a constant switching activity in the motor at a basal value despite fluctuations in the chemical environment. Continually adapting and maintaining a basal switching activity, measured as the fraction of time the motor rotates CW, helps the cell retain the ability to respond to novel stimuli. Thus, adaptation is crucial for chemotaxis.

In addition to motility and chemotaxis, the flagellar motor has another function, termed mechanosensing (see Table 1 for glossary). Flagellar mechanosensing enables the cell to detect changes in its mechanical environment by sensing changes in the viscous resistance (viscous load) to the rotation of the flagellar motor (Lele et al., 2013). Mechanosensing appears to be crucial for the bacterium to sense its adhesion to solid surfaces (Hughes and Berg, 2017). In turn, the flagellum and other appendages such as the pili likely trigger gene regulatory changes or post-translational modifications that help the cell adopt surface-associated lifestyles such as swarming or biofilms (Kearns, 2010; Jones et al., 2015; Chawla et al., 2020; Webster et al., 2022). These regulatory changes and surface-related phenotypes have been reviewed elsewhere (Belas, 2014; Laventie and Jenal, 2020; Wong et al., 2021). Recent work has identified an intimate link between flagellar mechanosensing, adaptation, and chemotaxis (Antani et al., 2021a). Here, we discuss possible mechanisms for this coupling and how it likely gives rise to proprioception—which refers to the ability of an organism to sense its position and velocity in space—in bacteria. We will conclude with a brief note on the implications of bacterial proprioception for bacterial colonization of surfaces.

**TABLE 1:** Glossary.

Term	Definition
Viscous load	Fluid resistance to the rotation of the flagellum or physical obstruction of rotation due to the adhesion of the filament to a surface. A motor lacking the flagellar hook and the filament is under negligible viscous load irrespective of any changes in the extracellular environment of the cell (Chawla et al., 2020).
Mechanical stimulus	Change in the viscous load on the flagellar motor
Mechanosensing	Adaptation/changes in protein function induced by mechanical stimulus
CW_bias_	Fraction of time the motor rotates CW
Torque	Force applied by a stator unit on the FliG ring, which induces the latter to rotate.
Reversal frequency	Number of switches between CW and CCW directions of rotation per unit time (usually per second)
Stalled motor	A motor that is unable to rotate because the resistance to its rotation exceeds the maximal torque it can generate.
Ultra-sensitivity curve	Sigmoidal relationship between CW_bias_ and CheY-P, characterized by a Hill coefficient ∼ 10–20.
Precise adaptation	Restoration of a function after a stimulus to its exact pre-stimulus value.

### Flagellar Switching and Torque

**FIGURE 1:**
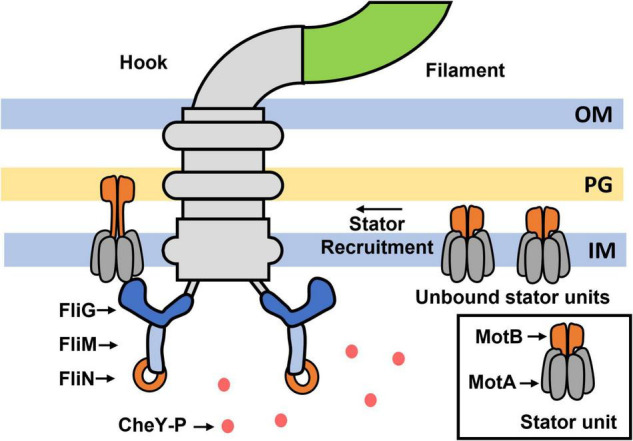
The flagellar motor. Major parts of the flagellar motor are indicated. Unbound stator units diffuse in the membrane and reversibly bind to the motor to induce rotation. The motor recruits additional stator units when the resistance to its rotation is increased (Lele et al., 2013). The FliG ring within the rotor interacts with the stator units, and its conformation determines the direction of rotation. Freely diffusing CheY-P binds to the FliM and FliN complexes located beneath the FliG ring to promote conformational changes in FliG.

The flagellum consists of an extracellular filament connected to a transmembrane rotary motor by a hook that serves as a universal joint (Figure 1). The flagellar motor consists of a rotor and a stator; the latter delivers torque to the former to induce rotation (Berg, 2004). Torque is generated by multiple stator units that may associate and dissociate from the motor as a function of several factors (Muramoto et al., 1994; Fung and Berg, 1995; Berg, 2003; Leake et al., 2006; Blair et al., 2008; Paulick et al., 2009; Lin et al., 2021). To generate torque, the stator units typically utilize the proton-motive force, although alternate sources of ion-motive force also may be used (Manson et al., 1977; Yorimitsu and Homma, 2001; Wilhelms et al., 2009; Terahara et al., 2012; Minamino and Imada, 2015; Imazawa et al., 2016). The stator complex functions as a mechanosensor by sensing changes in the viscous resistance to the rotation of the motor—also known as the viscous load—and adapts structurally and functionally in response to increased load (Lele et al., 2013; Chawla et al., 2017). The direction of motor rotation is determined by the conformations of the ring of FliG proteins within the flagellar rotor, which forms the track along which the stator units operate (Figure 1). The FliG ring consists of multiple subunits (34 in *E. coli*) (Lee et al., 2010). When every FliG subunit adopts the same conformation, the motor rotates at the maximum possible speed in a given direction for a particular viscous load (Bray and Duke, 2004). The FliG ring switches stochastically between two conformations, one favoring CCW rotation and the other favoring CW rotation. CheY-P binds to FliMN to stabilize the CW conformation of the FliG ring (Sarkar et al., 2010; Minamino et al., 2011, 2019).

Switching activity is quantified by the CW_bias_, which refers to the fraction of time the motor rotates CW. The CW_bias_ depends ultra-sensitively on CheY-P levels: the motor exhibits the entire range of CW_bias_ from 0 to 1 with an ∼1 μM change in intracellular CheY-P levels (Cluzel et al., 2000; Korobkova et al., 2006; Tu, 2008). The cell must maintain a steady, intermediate value of the basal bias (0 < CW_bias_ < 1), otherwise it cannot respond to chemical signals (Antani et al., 2021b). As motor reversals are inherently stochastic, the direction of rotation may change multiple times unpredictably in a second. The reversal frequency vs. CheY-P relationship is unimodal or bell-shaped (Cluzel et al., 2000), which means that there is no unique value of the reversal frequency with respect to the CheA activity. Hence, it is easier to interpret the response of the chemotaxis network to chemical stimuli from changes in CW_bias_ (Antani et al., 2021b).

Biophysical characterization of the flagellar motor typically involves monitoring the rotational direction and speeds of a latex bead attached to it. The viscous load on the motor is varied by using beads of different sizes. In *E. coli*, such experiments showed that variations in the viscous load alter the reversal frequency even when no chemical stimulus is present (Fahrner et al., 2003; Yuan et al., 2009a). The viscous resistance to rotation (load) only exists in the presence of torque—a large and a small latex bead do not represent significantly different loads in the absence of torque. The torque delivered by each stator unit increases with the viscous load (Ryu et al., 2000), which indicates that the reason the reversal frequency is load-sensitive is because torque influences the conformations of the FliG subunits.

How might torque influence FliG conformations? One possibility is that torque influences the activation barriers for FliG to switch between the CW and CCW conformations (Yuan et al., 2009a). This can cause each FliG subunit that comes in contact with a stator unit, as the rotor turns, to flip between CW and CCW conformations more or less frequently as a function of the torque experienced and of the duration of contact between the stator units and FliG subunits (Bai et al., 2012). But, these and other models offer limited quantitative insights as they assume that a constant number of stator units engage with the rotor irrespective of the load. In other words, the number of FliG subunits simultaneously experiencing torque under low and high loads are assumed to be the same (Bai et al., 2012).

**FIGURE 2:**
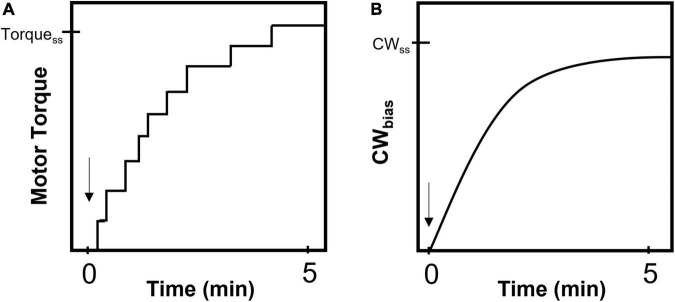
Mechano-response in a flagellar motor. Cartoon representations of the response of a single motor that is mechanically stimulated by suddenly increasing the viscous load (Lele et al., 2013). The arrow indicates the instant of stimulus. **(A)** The motor responds by recruiting additional stator units resulting in a stepwise increase in torque (and speed). Torque_SS_ indicates the final, steady-state value of torque following the completion of stator recruitment and can range from 2,000 to 4,000 pN.nm under high loads (Berg, 2004). **(B)** The probability of CW rotation (CW_bias_) increases from a very low value to a steady-state or basal value (CW_SS_) over a similar timescale as that for stator recruitment. The CW_SS_ value remains constant irrespective of the magnitude of the load-change (Antani et al., 2021a).

This assumption was invalidated when it was observed that the number of stator units associated with the motor increases with the viscous load: the motor recruits ∼1 stator unit under very low viscous loads and as many as 8–11 stator units under very high viscous loads (Figure 1; Lele et al., 2013; Tipping et al., 2013). When the viscous load on a motor that was rotated by a single stator unit was suddenly increased, torque increased in response and switching was inhibited. As the motor rotated predominantly CCW, this suggests that increased torque inhibits changes in the conformation of FliG from CCW to CW. Recent observations are consistent with this idea – in cells in which stator proteins are under-expressed, motors stably rotated by 1–2 stator units under high loads rotated mostly CCW (low CW_bias_) even when the CheY-P pool was above the native levels (Antani et al., 2021a). A key feature of flagellar mechanosensing is that additional stator units are gradually recruited to the motor under high loads, increasing the overall torque on FliG (Figure 2A). Interestingly, the CW_bias_ also increases as new stator units are recruited, suggesting that the switching activity adapts to variations in torque (Figure 2B; Lele et al., 2013). If increased torque applied by each stator unit inhibits changes in conformations of FliG subunits from CCW to CW, how do motors increase their CW_bias_ under high loads?

Models are evaluated based on their ability to accurately predict load-dependent variations in several characteristic features of flagellar switching: the reversal frequencies, the ultrasensitive dependence of CW_bias_ on CheY-P levels, and the wait-time distributions for CW and CCW rotation. Models invoking non-equilibrium mechanisms have explained the non-exponential distributions that have been observed for the time-intervals for CW or CCW rotation. Subsequent measurements of the interval distributions under near zero to high loads at various values of the proton motive force (PMF), and for different number of stator units bound to the motor, are all consistent with non-equilibrium mechanisms that involve some effect of torque on the probability of conformational changes in FliG (Wang et al., 2017). Recent measurements of switching under very high viscous loads, which almost prevent motor rotation, are also consistent with a model that attributes differential effects of load on switching to the asymmetry in torque experienced by FliG in the CW and CCW conformations (Yuan et al., 2009b; Wang et al., 2021).

To determine the mechanism by which constant CW_bias_ is maintained at high loads, Antani and co-workers imaged the binding of fluorescently labeled CheY-P to motors in tethered cells using total internal reflection fluorescence microscopy (Antani et al., 2021a). The tethered cell assay involves adhering a single flagellar filament to a glass substrate, which causes the cell body to rotate around the motor of interest (Silverman and Simon, 1974). In such motors, the authors observed that CheY-P binding was maximal when motors rotated with a full complement of stator units. In the absence of stator units, CheY-P binding was weaker (Antani et al., 2021a). This suggested that there is a proton flux-dependent mechanism of CheY-P binding as there is no significant flux of protons in motors lacking stator units. However, when optical traps were used to block the rotation of a tethered cell to inhibit proton flux, no inhibition in CheY-P binding was observed. In such stalled motors, the stator units remain engaged and continue to deliver torque (Tipping et al., 2013). Thus, it is not the change in proton flux but changes in torque that induced differential CheY-P binding (Antani et al., 2021a). Based on these findings, we proposed a model in which increased torque interferes with the conformational change in FliG from CCW to CW. However, increased torque also promotes CheY-P binding. This mechanosensitive binding of CheY-P appears to compensate for the inhibitory effects of torque on switching to CW rotation.

### Torque-Dependent Mechanisms of Stator Recruitment and CheY-P Binding

Experiments in *E. coli* suggest that there is a pool of ∼100 stator units within the cell membrane (Leake et al., 2006). Each stator unit consists of a pentamer of MotA and a dimer of MotB, forming proton channels that remain blocked by two plugs that prevent the leakage of protons into the cytoplasm from the periplasm (Hosking et al., 2006). When MotA interacts with FliG, the plugs open and interact with one another to allow the flow of protons (Hosking et al., 2006). This may enable relative motion between the MotB and the MotA interfaces; as per latest models, the relative motion involves the rotation of the MotA pentamer around the MotB dimer (Deme et al., 2020; Santiveri et al., 2020). This relative motion can transmit force to contacting FliG subunits resulting in a torque that rotates the motor. However, for proper transmission of the force to FliG and for the plugs to persist in the open position, the stator unit must be anchored in the cell wall (Zhu et al., 2014). Anchoring is achieved by extending the peptidoglycan binding domain (PGB) in MotB such that it associates with the cell wall (Van Way et al., 2000; Kojima et al., 2009, 2018).

There are numerous C-ring assemblies in the cell membrane that are not necessarily parts of functional motors (Delalez et al., 2010; Li and Sourjik, 2011). Co-isolation assays showed weak interactions between MotA and FliG (Tang et al., 1996), so the latter does not need to be a part of a fully functioning motor to interact with a stator unit. If stator-FliG interactions occur frequently in the membrane, what prevents stator units from conducting non-productive transmembrane proton flow? Probably, the PGB fail to anchor stably during such interactions. Hence, the opening of the plugs would be short-lived. Consequently, the stator units may simply diffuse away rather than continuously interacting with the pre-assembled C-rings. We propose that the reason the PGB does not anchor stably is because the FliG rings in pre-assemblies offer negligible viscous resistance for the stator units to work against – without the flagellar hook and a filament, the pre-assembled structure is always under negligible load irrespective of the viscosity of the extracellular environment (Chawla et al., 2020).

There is support for the idea that the strength of the association between the PGB and the cell wall increases with load. First, high loads induce higher torque from the stator units (Ryu et al., 2000), which indicates that there is a correlation between high loads and stable association between the PGB and the cell wall. Second, paralyzed or defective stator units with mutant MotA subunits exhibit weak association with motors, as seen in tethered cells, likely because the PGB fails to anchor properly in these mutants (Chawla et al., 2017). Finally, experimental observations are consistent with a model in which the dissociation rate of a stator unit from the motor decreases with an increase in the torque it delivers (Chawla et al., 2017). Thus, application of torque to FliG requires stator anchoring within the cell wall and increases the strength of that attachment, which potentially explains how mechanosensitive recruitment of stator units to the motor occurs (Lele et al., 2013; Nord et al., 2017; Terahara et al., 2017). Interested readers are referred elsewhere for a detailed theoretical exposition of the torque-dependent stator binding (Wadhwa et al., 2019).

Once MotB anchors and the stator unit begins delivering torque, Newton’s third law dictates that an equal and opposite (reactive) torque must simultaneously act on the interface between the PGB and the peptidoglycan (Antani et al., 2021a). The notion of a reactive torque acting on the PGB is consistent with the notion that the stator unit itself is a rotary motor (Chang et al., 2020; Deme et al., 2020; Santiveri et al., 2020). The reactive torque could strengthen the association of the PGB with the cell wall by creating a torsional twist within the stator unit, thereby uncovering additional peptidoglycan-binding sites within PGB (Chawla et al., 2017) or by activating a mechanosensitive component within MotB that stabilizes the extended conformation of the PGB (Chawla et al., 2017; Nord et al., 2017). We propose an alternate basis for the mechanosensitive association of the stator units with the cell wall. The torsional twist could embed the PGB within the peptidoglycan, like a fork spinning in spaghetti, strengthening the association between the PGB and peptidoglycan. The entanglement of the PGB in the peptidoglycan is likely stronger when the stator unit delivers higher torque, causing a decrease in the dissociation rate and an increase in the dwell time of the stator unit at the motor. There is only partial cross-linking within the peptidoglycan (Glauner, 1988; Glauner et al., 1988; Meroueh et al., 2006) and the pore-size of the cell wall is similar to the dimensions of the PGB (Meroueh et al., 2006; Roujeinikova, 2008), suggesting that the cell wall is flexible locally and could support torque-dependent entanglement of the PGB.

The proposed mechanism does not require the presence of a mechanosensitive domain within the stator unit, and it is consistent with a strong chemical affinity between the PGB and the peptidoglycan (Roujeinikova, 2008). In some bacterial species, such as *Pseudomonas aeruginosa*, which carries more than one type of stator, the viscous load modulates competitive docking of stator units at the motor (Wu et al., 2021). The outcome of the competition between different stator types will be determined by differences in the torsional rigidity of stator components, the rigidity of the peptidoglycan network, the amount of torque each stator type can generate against a particular load, the ionic strength, and the relative affinities of the different PGB domains for the cell wall. Several types of regulators also interact with the stator and/or the rotor to modulate torque (Subramanian and Kearns, 2019). These regulators may affect the load-dependence of the association of the PGB with the cell wall to influence mechanosensitive stator recruitment.

Although the predicted effects of the reactive torque on the interactions of the PGB with the cell wall are yet to be tested, in *E. coli* the torque on FliG has recently been shown to affect CheY-P interactions with the motor. The force delivered to FliG strengthens the binding of CheY-P to FliM and FliN complexes at the base of the motor although the binding sites are almost 15 nm away from the site of torque delivery (Figure 1). An allosteric mechanism is likely involved, but details are lacking (Antani et al., 2021a). It is possible that small conformational shifts induced in FliG because of increasing torque might cause downstream conformational changes in FliM or FliN to increase their affinity for CheY-P. The exact mechanism is unknown; a complication is that the affinity of FliM/FliN for CheY-P is lower when FliG is in its CCW conformation (Fukuoka et al., 2014). As the increased torque increases the probability that FliG adopts the CCW conformation, CheY-P binding is predicted to decrease as more stator units are recruited following a load increase, contrary to observations. Nevertheless, the mechanosensitive nature of CheY-P binding suggests that chemotaxis and flagellar mechanosensing are coupled.

### Mechanosensitive CheY-P Binding and Precise Adaptation

**FIGURE 3:**
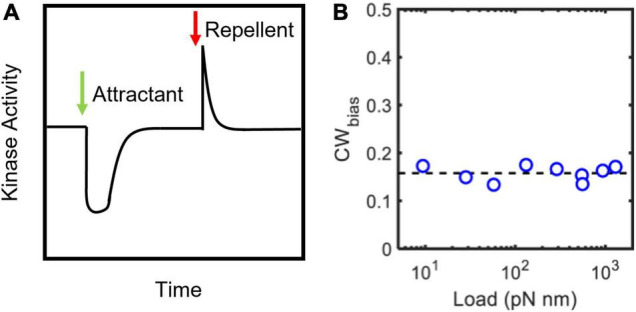
Precise adaptation in chemotactic activity. **(A)** Cartoon representation of CheA responses to step changes in ligand levels (Sourjik and Berg, 2002). CheA activity was calculated from measurements of Förster resonance energy transfer (FRET) between CheY-eYFP and CheZ-CFP in live cells (Sourjik and Berg, 2002). The addition of an attractant (green arrow) or repellent (red arrow) decreases or increases the kinase activity, respectively. CheR and CheB help adapt the activity precisely to its basal value. Consequently, CheY-P levels and CW_bias_ (not shown) also precisely adapt. **(B)** The steady-state CW_bias_ is independent of the load (Yuan et al., 2009a; Antani et al., 2021a). This observation suggests that the adaptation of the CW_bias_ to mechanical stimuli (shown in Figure 2B) is precise. The adaptation is network independent.

The chemotaxis network is highly sensitive to extracellular ligands over a wide range of concentrations (Berg, 2004). Chemical signals sensed by the chemoreceptors are greatly amplified to modulate the flagellar switch response. To avoid saturating a system with such high gain, CheR and CheB help adapt the kinase activity to keep CheY-P levels at the basal value (Figure 3A; Sourjik and Berg, 2002). This adaptation at the input of the chemotaxis network is rapid, typically occurring over a few seconds (Segall et al., 1982). Such short-time adaptation maintains a basal level of flagellar switch activity (CW_bias_) that ensures that the cell can respond to novel chemical stimuli and continue swimming along a gradient of ligands (Berg and Purcell, 1977). There is no evidence that chemoreceptors respond to mechanical feedback from the motor (Shimizu et al., 2006). As mechanical stimuli can inhibit switching (Figure 2B), the flagellar motor must find a way to adapt to changes in viscous load, failing which the cell will lose its ability to perform chemotaxis in environments with widely different viscosities.

Yuan et al. (2012) discovered that the motor adapts to changes in CW_bias_ induced by chemical stimuli. The FliM and FliN complexes in *E. coli* can remodel to offset long-term fluctuations in CheA activity. FliM/FliN remodeling probably occurs because FliG subunits bind to FliM and FliN subunits with a higher affinity when the motor is in the CCW conformation compared to the CW conformation (Lele et al., 2012). For example, a long-lived decrease in the CheA activity, leading to lower CheY-P levels, induces FliG subunits to adopt the CCW conformation, thereby decreasing the CW_bias_. This causes the number of FliM/FliN subunits bound to the motor to increase, presumably helping the motor bind more CheY-P. What follows is a partial adaptation in the CW_bias_. Later work indicated that FliM/FliN remodel each time the motor stochastically switches between the CCW and CCW directions irrespective of the CheA activity, with the number of FliM/FliN subunits added or removed increasing with the duration of the CCW or CW interval, respectively (Lele et al., 2015; Liu et al., 2020). FliM/FliN remodeling does not promote precise adaptation in CW_bias_ but does appear to complement and accelerate chemoreceptor-mediated adaptation for optimizing chemotaxis (Dufour et al., 2014; Zhang et al., 2018).

**FIGURE 4:**
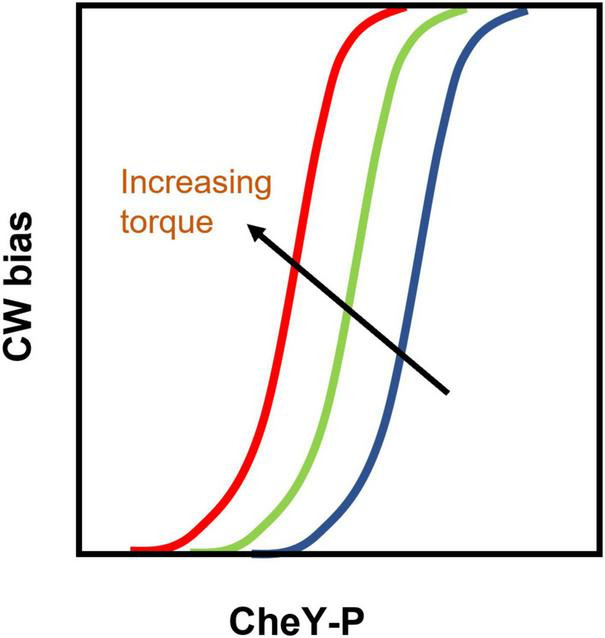
Tuning of the CW_bias_ –CheY-P relationship by torque: Cartoon representation of the analysis of experimental results (Antani et al., 2021a). Increasing torque shifts the CW_bias_ versus [CheY-P] curve leftward (from the position indicated by the blue curve to the one indicated by the red curve) by modulating the affinity of FliM/FliN for CheY-P. As a result, the CW_bias_ increases with increasing torque even though CheY-P levels remain constant. Thus, the shifting curves compensate for the inhibitory effects of increased viscous load on the motor and help maintain a load-independent CW_bias_ (shown in Figure 3B).

The basal value of CW_bias_ is independent of load (Figure 3B), despite the inhibitory effects of load on switching (Figure 2B). This suggests that the CW_bias_ adapts precisely any time there is a change in the load. Do FliM and FliN remodel to enable precise adaptation in switching in response to such mechanical stimuli? Experiments have ruled out this possibility (Antani et al., 2021a). Instead, the motor precisely adapts by modulating the affinity of FliM/FliN for CheY-P following the mechanical stimulus. These changes in affinity fine-tune the dependence of the CW_bias_ on CheY-P, a relationship characterized by a steep sigmoidal curve (Cluzel et al., 2000). Experiments indicate that the tuning mechanism involves shifts in the CW_bias_–CheY-P curve with varying torque, as shown in Figure 4, increasing or decreasing the sensitivity of the motor for CheY-P (Antani et al., 2021a). An undescribed feedback mechanism must be required for such precision. In addition to torque-dependent affinity for CheY-P, the duration of the contact between each FliG subunit and the stator likely plays a key role in the feedback as it may affect the duration of mechanosensitive CheY-P binding to the FliM/FliN subunits in contact with that FliG subunit. The time each stator unit and FliG are in contact depends on the rotation rate (Bai et al., 2012), and the rotation rate determines the swimming speed. Hence, the swimming speed is indirectly expected to influence the feedback. It is possible, therefore, that this mechanism enables adaptation to changes in the swimming speed, as when the bacterium enters an environment of a different viscosity.

### Bacterial Proprioception

The tendency of the flagellum to maintain a constant switching activity under varying viscous loads is likely to be critical for allowing peritrichous bacterial species such as *E. coli* to run and tumble even as new flagella are being assembled. As a new flagellum is being formed, the filament length initially is very short, and the new motor experiences a low load. As the filament grows, the motor experiences an increasing viscous load. Without the tuning of the sensitivity curves (Figure 4), the growing filaments will cease to switch once the filament reaches a certain length. The same principle will apply to polarly flagellated species; as the filaments grow, the polar motors must adapt to the increasing load to continue performing runs and reversals. Therefore, mechanosensitive binding of CheY-P is likely a widespread phenomenon.

The tuning of sensitivity to stimuli in response to mechanical stress, such as the one seen in Figure 4, is common in higher organisms. For example, proprioceptive feedback in the motor neurons that enervate the leg muscles in insects helps maintain maximal sensitivity to different mechanical loads. This allows the organism to maintain posture and grip when walking on the floor or the ceiling. More broadly, proprioception refers to an organism’s ability to sense its movements and/or position in space (Tuthill and Azim, 2018; Harris et al., 2020). A familiar example would be a soccer player judging how fast a ball is traveling to intercept it precisely in three dimensions. Bacteria lack sophisticated sensory systems and a central nervous system. Nonetheless, the coupling between the mechanosensitive stators, CheY-P, and the output of the chemotaxis system provides bacteria with what are essentially proprioceptive abilities.

Bacterial proprioception probably helps the cell sense its own position relative to a surface and its adhesion to the surface. It also enables chemotaxis when cells encounter highly viscous environments such as the mucous layers coating the intestine or gel-like media. Thus, the cell can adapt its flagellar functions to continue chemotaxis, which is important for surface colonization (Tamar et al., 2016).

However, the limits of mechanosensitive adaptation may be exceeded in certain scenarios. For example, in swarming colonies, CheY-P levels are so low that the probability of switching is significantly diminished despite any adaptations (Ford et al., 2018; Partridge et al., 2019). We speculate that such a loss in switching may trigger downstream signaling events to sustain the swarming state of the colony. Although stator mechanosensing and mechanosensitive CheY-P binding were the focus in this review, it is possible that a similar proprioceptive coupling exists between stators and other functional regulators of the motor. In that case, proprioception might regulate numerous other developmental effects, including biofilm formation, possibly by modulating secondary messenger levels (Boyd and O’toole, 2012; Webster et al., 2022).

## Author Contributions

RG and PL wrote the manuscript with inputs from JY. All authors approved it for publication.

## Conflict of Interest

The authors declare that the research was conducted in the absence of any commercial or financial relationships that could be construed as a potential conflict of interest.

## Publisher’s Note

All claims expressed in this article are solely those of the authors and do not necessarily represent those of their affiliated organizations, or those of the publisher, the editors and the reviewers. Any product that may be evaluated in this article, or claim that may be made by its manufacturer, is not guaranteed or endorsed by the publisher.
